# Study on the Microstructure and Properties of Plasma-Sprayed HA/ZrO_2_/Nb Composite Coatings with Varying Nb Contents

**DOI:** 10.3390/ma19214541

**Published:** 2026-10-28

**Authors:** Jiaqi Li, Liming Qian, Xiaofeng Wan, Xiang Li, Rui Wang

**Affiliations:** 1 School of Mechanical Engineering (School of Intelligent Manufacturing), Nantong Institute of Technology, Nantong 226002, China; 2 School of Mechanical Engineering, Nantong University, Nantong 226019, China; 3 School of Mechanical, Materials, Mechatronic and Biomedical Engineering, University of Wollongong, Wollongong, NSW 2522, Australia

**Keywords:** magnesium alloy, HA/ZrO_2_/Nb composite coating, atmospheric plasma spraying, corrosion resistance, cytocompatibility

## Abstract

Magnesium alloys are promising biodegradable implants, but rapid degradation and insufficient early-stage bioactivity limit their clinical use. In this study, hydroxyapatite (HA)/zirconia (ZrO_2_)/niobium (Nb) composite coatings with varying Nb contents (10, 20, and 30%) were deposited on ZK60 magnesium alloys via atmospheric plasma spraying. The coatings retained feedstock phases while forming tetracalcium phosphate, tricalcium phosphate, and Nb_2_O_5_ during spraying. Microstructurally, increasing Nb to 30% induced an interconnected porous network, resulting in increased surface microhardness (~426 HV) and hydrophilicity (contact angle ~50°). Electrochemical tests revealed that, among the investigated compositions, the 20% Nb coating exhibited the lowest corrosion current density (I_corr_ = 9.688 × 10^−5^ A/cm^2^), indicating the most favorable electrochemical behavior within the tested Nb-content range. Although further increasing Nb to 30% resulted in a higher I_corr_ than that of the 20% Nb coating, H1Z3N exhibited the highest day-5 OD450 value and favorable cell spreading. This cellular response may be associated with its higher surface roughness, enhanced wettability, and porous architecture. Overall, the investigated compositions exhibited an observed trade-off between electrochemical behavior and cytocompatibility.

## 1. Introduction

As a vital organ and tissue in the human body, bone plays a crucial role in providing mechanical support, protecting internal organs, and storing minerals [1,2]. With the aging population and the increasing incidence of traumatic events, the clinical demand for bone implant substitute materials has risen sharply [3,4]. Traditional metallic implants, such as titanium alloys, stainless steels, and cobalt–chromium alloys, possess extremely high mechanical strength; however, their elastic moduli are higher than those of natural human bone. This mismatch can easily induce the “stress shielding” effect, subsequently leading to osteoporosis and aseptic loosening around the implants [5]. Furthermore, these inert metals require a secondary surgical procedure for removal after bone healing, which increases patient suffering and medical costs. In contrast, magnesium (Mg) and its alloys exhibit excellent biomimetic mechanical properties due to their density (1.7–1.9 g/cm^3^) and elastic modulus (41–45 GPa) being extremely close to those of natural bone tissues [6,7]. More importantly, Mg is an essential macroelement in the human body, featuring excellent biocompatibility and in vivo biodegradability, and is thus hailed as a revolutionary “green” biomedical implant material [8,9,10]. However, Mg-based implants are highly susceptible to rapid corrosion and degradation in complex physiological environments (which are rich in water, chloride ions, etc.). An excessively fast degradation rate not only causes the implant to lose mechanical integrity before the bone tissue is fully healed, but also releases a large volume of hydrogen gas and causes local microenvironmental alkalization during the degradation process. This can trigger inflammatory responses and even lead to tissue necrosis, severely restricting their widespread clinical application [11,12,13,14].

To address the rapid in vivo degradation of Mg alloys, surface modification technology is considered one of the most effective strategies to enhance their corrosion resistance and surface bioactivity [15,16]. Chen et al. [17] systematically reviewed the latest advances in surface engineering techniques for biodegradable magnesium alloy orthopedic implants, demonstrating that composite ceramic coatings can simultaneously achieve degradation rate regulation and surface bioactivity optimization, and have emerged as the mainstream technical direction for surface modification of magnesium implants. Kumar et al. [18] systematically summarized the fabrication strategies and research challenges of hydroxyapatite coatings on biodegradable magnesium alloys, pointing out that the synergistic regulation of plasma spraying process parameters and coating composition is the core factor determining the long-term protective performance and bioactivity of the coatings. Among various modification materials, hydroxyapatite (HA, Ca_10_(PO_4_)_6_(OH)_2_) is widely used as a surface coating for orthopedic implants due to its high chemical similarity to natural human bone minerals, exhibiting excellent osteoconductivity and osteoinductivity [19,20]. Among numerous coating preparation technologies, such as atmospheric plasma spraying (APS), sol–gel, and electrodeposition, APS has become the mainstream method for fabricating commercialized medical bioceramic coatings and has been approved by the US Food and Drug Administration (FDA), owing to its high deposition efficiency, strong bonding strength, and low operational cost [21,22]. Nevertheless, plasma-sprayed pure HA coatings also face several challenges. On the one hand, HA possesses inherently high brittleness and poor fracture toughness; on the other hand, under the extremely high temperature gradients typical of thermal spraying, HA powders are prone to dehydroxylation and thermal decomposition. This generates amorphous phases or highly soluble byproducts, such as tricalcium phosphate (TCP), tetracalcium phosphate (TTCP), and even calcium oxide (CaO). These defects lead to the rapid dissolution of the coating in body fluids, triggering coating cracking and peeling, thereby weakening its long-term protective capability for the Mg substrate [23,24,25,26]. Recent advances have highlighted the necessity of composite surface architectures to enhance barrier protection and biological properties on magnesium alloys. For example, Kumar et al. [27] developed Fe/Ni/Cr oxide nanoparticles/PLA hybrid composite coatings on a Mg-Zn-Ca alloy, discovering that the hybrid structure significantly reduced the degradation rate, which proved its effectiveness in providing sustainable mechanical integrity. Furthermore, Feng et al. [28] fabricated a gallium-coated ZK60 magnesium alloy, finding that the protective barrier exhibited superior corrosion resistance and favorable antimicrobial properties, which proved the potential of multifunctional coatings in promoting osteogenesis while controlling premature dissolution.

To overcome the mechanical and chemical instabilities of pure HA coatings, the introduction of second-phase materials (such as metals or other oxide ceramics) to construct composite coatings has become a primary research focus [29]. Various oxide ceramics, including TiO_2_ and Al_2_O_3_, have also been investigated as protective coatings for biodegradable magnesium alloys due to their excellent chemical stability and mechanical properties. However, TiO_2_ generally exhibits limited bioactivity, while Al_2_O_3_ is considered relatively bioinert, which may restrict their application in bone-related implants. Previous studies have reported that the incorporation of metal oxides into HA coatings can improve their comprehensive performance. For example, HA/TiO_2_ composite coatings have been investigated for enhanced corrosion protection and structural stability, while HA/Al_2_O_3_ composite coatings have been explored to improve mechanical strength and wear resistance. These studies demonstrate the potential of oxide reinforcement in overcoming the intrinsic limitations of pure HA coatings. Zirconium dioxide (ZrO_2_) is widely used in the biomedical field due to its extremely high compressive strength and excellent biocompatibility. More importantly, the addition of stabilized ZrO_2_ can undergo a phase transformation from the tetragonal phase (t-ZrO_2_) to the monoclinic phase (m-ZrO_2_) under the stress field at the crack tip. The volume expansion and shear strain generated by this phase transformation can effectively inhibit the propagation of microcracks, thereby achieving transformation toughening of the coating [30,31,32]. In addition to oxide reinforcement, metallic elements have also been incorporated into HA coatings to improve their mechanical and biological performance. For example, Ti-, Mg-, and Zn-containing HA composite coatings have been investigated due to their favorable mechanical properties, biodegradability, or biological activity. However, some metallic reinforcements may suffer from rapid degradation, insufficient long-term stability, or potential concerns regarding excessive ion release. Therefore, selecting an appropriate metallic component is critical for achieving a balance between mechanical reinforcement, corrosion protection, and biological compatibility. Meanwhile, metallic niobium (Nb), as a highly promising biophilic transition metal, possesses outstanding corrosion resistance, good ductility, and extremely low biotoxicity, and has been proven to promote fibroblast growth and osseointegration [33,34]. Verma et al. [35] stated in their latest review that the introduction of niobium and its oxides as reinforcing phases into HA-based coatings can simultaneously improve the mechanical strength, corrosion barrier performance and biological activity of the coatings, serving as an important approach to optimize the overall performance of HA-based biocoatings; however, the effects of systematically varying Nb content in HA-based coatings on magnesium alloy substrates remain insufficiently understood. Previous studies have also indicated that the Nb_2_O_5_ ceramic phase, formed by the partial oxidation of Nb during plasma spraying, can increase the surface energy of the material, facilitating the growth of hydroxyapatite crystals and the adhesion and proliferation of bone-related cells [36,37,38].

Our previous work [39] showed that the incorporation of Nb and ZrO_2_ into HA-based coatings can improve the microstructure, corrosion resistance, and cytocompatibility of coatings on ZK60 Mg alloy. However, that study considered only a single Nb content and therefore did not systematically evaluate the effect of varying Nb content [40]. In addition, a recent related study [41] investigated the effects of plasma-spraying power and powder feeding rate on HA/Nb/ZrO_2_ composite coatings and identified 33 kW and 18 g/min as suitable processing parameters. The ZrO_2_ content was fixed at 10 wt.% based on our previous study, in which this concentration provided a favorable balance between coating integrity, corrosion protection, and biological performance. Therefore, ZrO_2_ was maintained at 10 wt.% in the present study to minimize the influence of ZrO_2_ variation and to systematically investigate the effect of Nb content. The present study differs from these previous studies by fixing the spraying conditions at 33 kW and 18 g/min and the ZrO_2_ content at 10 wt.%, while systematically varying the Nb content from 10 to 30 wt.%. The H1Z1N, H1Z2N, and H1Z3N specimens and the experimental data reported in the present study were independently prepared and obtained; no specimens or experimental data from the previous study were reused. In particular, the Ra value of 2.128 μm for H1Z1N was independently measured in the present experimental series.

Recent reviews have also indicated that most studies on Nb-reinforced HA coatings have focused on binary HA/Nb systems, while the effects of Nb content in HA/ZrO_2_/Nb ternary coatings on magnesium substrates remain insufficiently understood [35]. Therefore, the present study systematically evaluates the relationships between Nb content, coating microstructure and physicochemical properties, electrochemical behavior, and cellular response. Among the three investigated compositions, H1Z2N (20 wt.% Nb) exhibited the most favorable electrochemical behavior, whereas H1Z3N (30 wt.% Nb) showed the highest day-5 cellular response. These results indicate an observed trade-off between electrochemical behavior and cellular response among the investigated compositions.

## 2. Materials and Methods

### 2.1. Material and Deposition of Coatings

A ZK60 magnesium alloy was selected as the substrate material and cut into specimens with dimensions of 10 mm × 10 mm × 10 mm. The ZK60 magnesium alloy plate was purchased from Dongguan Yisheng Metal Materials Co., Ltd. (Dongguan, China). Commercially available powders with a purity of 99.9% were utilized. Hydroxyapatite (HA) powder was supplied by Beijing DK Nano Technology Co., Ltd. (Beijing, China), yttria-stabilized ZrO_2_ powder (ZrO_2_, 3 mol% Y_2_O_3_, purity ≥ 99.9%) was obtained from Shanghai Chaochun New Material Co., Ltd. (Shanghai, China), and niobium (Nb) powder was provided by Hunan Hengxin Hard Material Co., Ltd. (Loudi, China). The HA, ZrO_2_, and Nb powders exhibited spherical, spherical, and near-spherical morphologies, with nominal particle sizes corresponding to 400 mesh, 200 mesh, and 200 mesh, respectively. To investigate the influence of Nb substitution level on the performance of the composite coatings, HA was progressively replaced by Nb while maintaining the ZrO_2_ content at 10 wt.%. Therefore, the variation in Nb content represents a compositional adjustment involving both increased Nb fraction and decreased HA fraction. The specific sample designations and their corresponding weight compositions are summarized in Table 1. The weighed powders were placed into a planetary ball mill (LPB-0.4L, Hunan LAISDA Instrument Equipment Co., Ltd., Yueyang, China) and mixed using anhydrous ethanol as the milling medium. The rotation speed was set to 500 r/min, and the milling time was 10 h, with the rotation direction reversed every 15 min. Following the milling process, the mixed powders were dried in a blast drying oven (DHG-9070A, Shanghai Yiheng Scientific Instrument Co., Ltd., Shanghai, China) at 80 °C for 5 h and subsequently sieved to remove agglomerated particles and improve powder flowability before spraying. Prior to spraying, the ZK60 Mg alloy substrates were sequentially polished using silicon carbide (SiC) abrasive papers ranging from 200# to 1500#, and then polished to a mirror finish using 0.5 μm diamond paste. The substrates were ultrasonically cleaned in acetone, anhydrous ethanol, and deionized water using a KQ-250DE ultrasonic cleaner (Kunshan Shumei Ultrasonic Instrument Co., Ltd., Kunshan, Jiangsu, China), and then dried with cold air. To remove the passivation oxide scale and enhance the mechanical interlocking between the substrate and the coating, the specimen surfaces were sandblasted using 80# brown corundum. The sandblasting parameters were controlled as follows: air pressure of 0.5–0.6 MPa, blasting distance of 100–150 mm, and blasting angle of 60–75°. Atmospheric plasma spraying (APS) was performed using a DH-2080 plasma spraying system (Shanghai Dahao Ruifa Spraying Machinery Co., Ltd., Shanghai, China) to deposit the composite coatings onto the ZK60 substrates. Based on preliminary process optimization, the spraying parameters were fixed as follows: spraying power of 33 kW, powder feeding rate of 18 g/min, argon (Ar) as the primary gas, hydrogen (H_2_) as the auxiliary gas, nitrogen (N_2_) as the carrier gas, spraying distance of 100–120 mm, and a powder feeder rotation speed of 3.5 r/min.

### 2.2. Characterization of Coatings

The phase compositions of the powders and the sprayed coatings were analyzed using an X-ray diffractometer (XRD, Ultima IV, Rigaku Corporation, Akishima, Tokyo, Japan). The measurements were conducted with a Cu-Kα radiation source (40 kV, 30 mA) over a 2θ range of 10° to 90°, at a scanning rate of 5°/min and a step size of 0.02°. The surface and cross-sectional micro-morphologies, as well as the elemental distributions of the coatings, were observed and analyzed using a field emission scanning electron microscope (SEM, GeminiSEM 300, Carl Zeiss AG, Oberkochen, Germany) equipped with an energy-dispersive X-ray spectrometer (EDS).

The surface roughness (Ra) of the coatings was evaluated using a step-type surface roughness tester (JB-4C, Shanghai Taiming Optical Instrument Co., Ltd., Shanghai, China). Ten locations were randomly selected on the same plane for measurement, and the average value was calculated. The microhardness of the coatings was measured using a Vickers hardness tester (TMVS-1, Beijing TIME High Technology Ltd., Beijing, China) under a test load of 100 gf (0.98 N) with a dwell time of 15 s. Three independently sprayed specimens were prepared for each coating composition. For each specimen, ten random indentations were performed on the coating surface, and the average microhardness value was calculated. The final result was reported as the mean value obtained from three independent specimens. The surface wettability of the coatings was evaluated using a contact angle measuring instrument (OCA15EC, Beijing Aodelino Instrument Co., Ltd., Beijing, China). A 5 μL droplet of simulated body fluid (SBF) was deposited onto the specimen surface. After the droplet stabilized, the contact angle was recorded. Three independently prepared specimens were evaluated for each coating composition. For each specimen, contact angle measurements were performed at five randomly selected positions, and the average value was calculated. The results are presented as the mean ± standard deviation of three independent specimens.

### 2.3. Corrosion Behavior Analysis

The electrochemical corrosion resistance of the coatings was evaluated in a standard Kokubo SBF solution using an electrochemical workstation (CHI660C, Shanghai Chenhua Instrument Co., Ltd., Shanghai, China). The tests were performed using a standard three-electrode system, with a saturated calomel electrode (SCE; model 232, Shanghai INESA Scientific Instrument Co., Ltd., Shanghai, China) as the reference electrode, a platinum electrode as the counter electrode, and the cold-mounted coating sample with an exposed testing area of 1 cm^2^ as the working electrode. Before polarization measurements, the open-circuit potential (OCP) was monitored for at least 300 s in naturally aerated SBF at room temperature. No predefined numerical OCP stabilization criterion was applied. The potential variation during the final 60 s was approximately 1–6 mV among the investigated specimens. Potentiodynamic polarization measurements were conducted over sample-specific potential ranges covering both cathodic and anodic branches at a scan rate of 1 mV/s. The electrochemical test conditions were selected with reference to ISO 10993-15:2019 [42] and an established laboratory protocol, and identical conditions were applied to all specimens to enable comparative evaluation. The polarization data were reanalyzed using OriginPro 2022 by linear regression of selected anodic and cathodic regions. E_corr_ and I_corr_ were determined from the intersection of the corresponding regression lines, and the fitted slopes and R^2^ values are reported in Table 2.

### 2.4. Cytocompatibility Evaluation

In vitro cytocompatibility tests were conducted using rat bone marrow mesenchymal stem cells (BMSCs) to evaluate the biocompatibility of the coatings. The BMSCs were cultured under standard conditions at 37 °C with 5% CO_2_. The coated samples were sterilized in an autoclave at 121 °C for 15 min and then placed into 24-well plates. A BMSC suspension with a density of 1 × 10^4^ cells/well was inoculated into each well, supplemented with fresh culture medium, and incubated in a constant temperature incubator at 37 °C with 5% CO_2_. After culturing for 1, 3, and 5 days, cell proliferation was evaluated using the Cell Counting Kit-8 (CCK-8; CK04, Dojindo Laboratories, Kumamoto, Japan) colorimetric assay. Following the removal of dead cells by washing, the culture medium containing the CCK-8 reagent was added and incubated for 2 h. The optical density values at 450 nm (OD450) of each well were subsequently measured at a wavelength of 450 nm using a microplate reader (Multiskan FC, Thermo Fisher Scientific, Waltham, MA, USA). Three parallel samples were established for each experimental group to minimize errors. Additionally, the cell morphology, adhesion, and growth status on the sample surfaces were observed and recorded using an inverted fluorescence microscope (ECLIPSE Ti-s, Nikon Corporation, Tokyo, Japan).

## 3. Results and Discussion

### 3.1. Phase Composition of the Coatings

**Figure 1:**
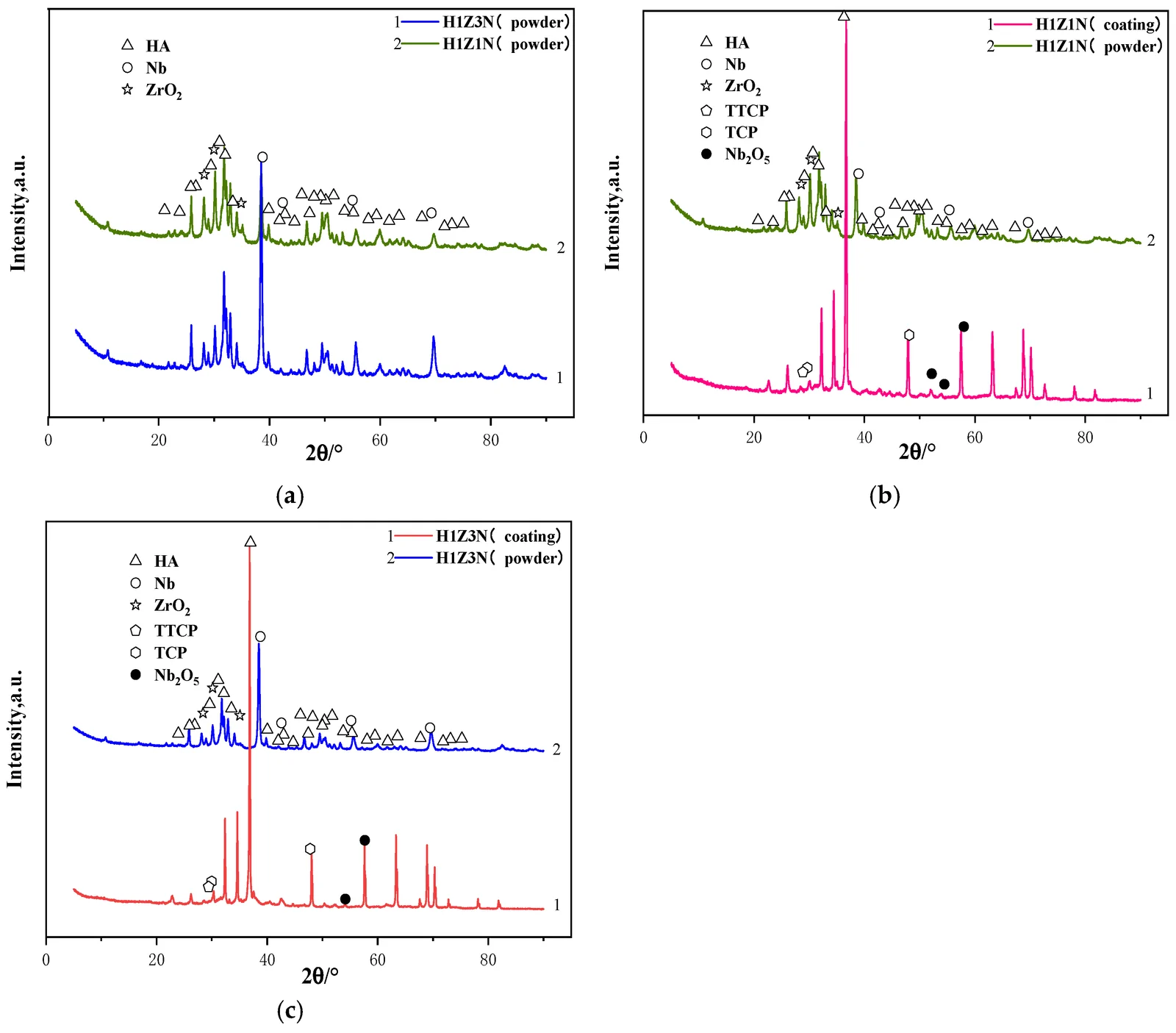
XRD patterns of the mixed powders and the as-sprayed composite coatings: (**a**) mixed powders of H1Z1N and H1Z3N; (**b**) H1Z1N powder and coating; (**c**) H1Z3N powder and coating.

The phase compositions of the mixed powders and the composite coatings with different Nb contents were analyzed using X-ray diffraction (XRD). The diffraction peaks were indexed according to the standard ICDD reference cards, including HA (PDF No. 09-0432), tetragonal ZrO_2_ (PDF No. 30-1468), and Nb (PDF No. 35-0789). As shown in Figure 1a, the feedstock powders mainly consisted of HA, ZrO_2_, and Nb phases without obvious impurity peaks, and the intensity of the Nb diffraction peaks varied correspondingly with the designed weight ratios. After the atmospheric plasma spraying process, the XRD patterns in Figure 1b,c reveal that the original HA, ZrO_2_, and Nb phases were successfully retained in the deposited H1Z1N and H1Z3N coatings. In addition to the original components, new diffraction peaks corresponding to tetracalcium phosphate (TTCP), tricalcium phosphate (TCP), and niobium pentoxide (Nb_2_O_5_) were detected in the sprayed coatings. The formation of TCP and TTCP is attributed to the thermal decomposition and dehydroxylation of HA caused by the ultra-high temperature of the plasma jet. Meanwhile, the Nb_2_O_5_ phase was formed due to the oxidation of metallic Nb interacting with oxygen during flight. As the Nb content increased from 10% to 30%, the types of generated decomposition or oxidation phases remained unchanged. However, compared to the H1Z1N coating (Figure 1b), the diffraction peaks in the H1Z3N coating showed higher intensity, which may be related to the increased Nb fraction and the variation in phase composition. It should be noted that quantitative phase analysis, FWHM analysis, and Rietveld refinement were not performed in this study; therefore, the effect of Nb content on the phase composition and structural characteristics of the coatings requires further investigation.

### 3.2. Microstructure and Elemental Distribution

**Figure 2:**
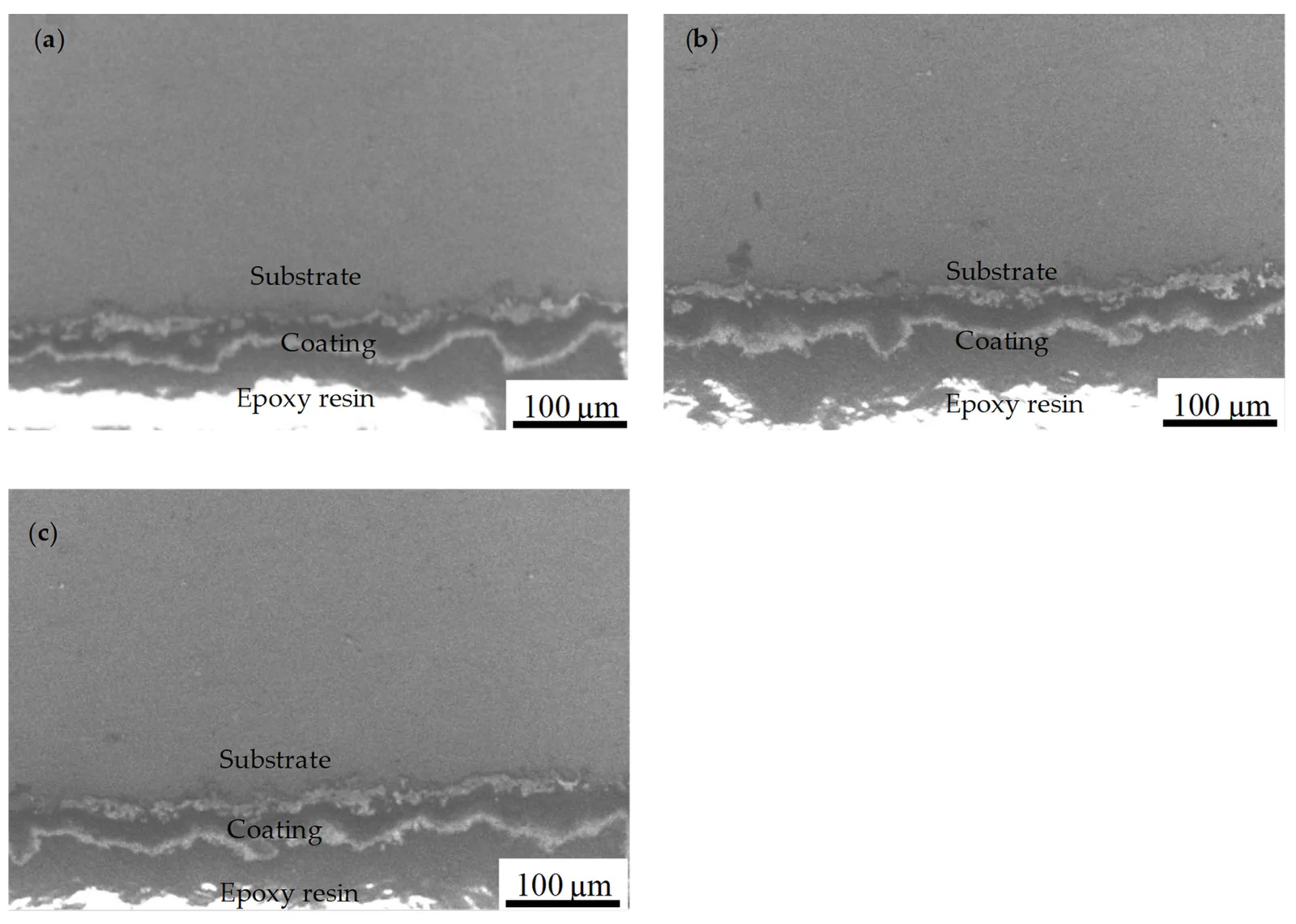
Cross-sectional SEM images of the composite coatings: (**a**) H1Z1N; (**b**) H1Z2N; (**c**) H1Z3N.

**Figure 3:**
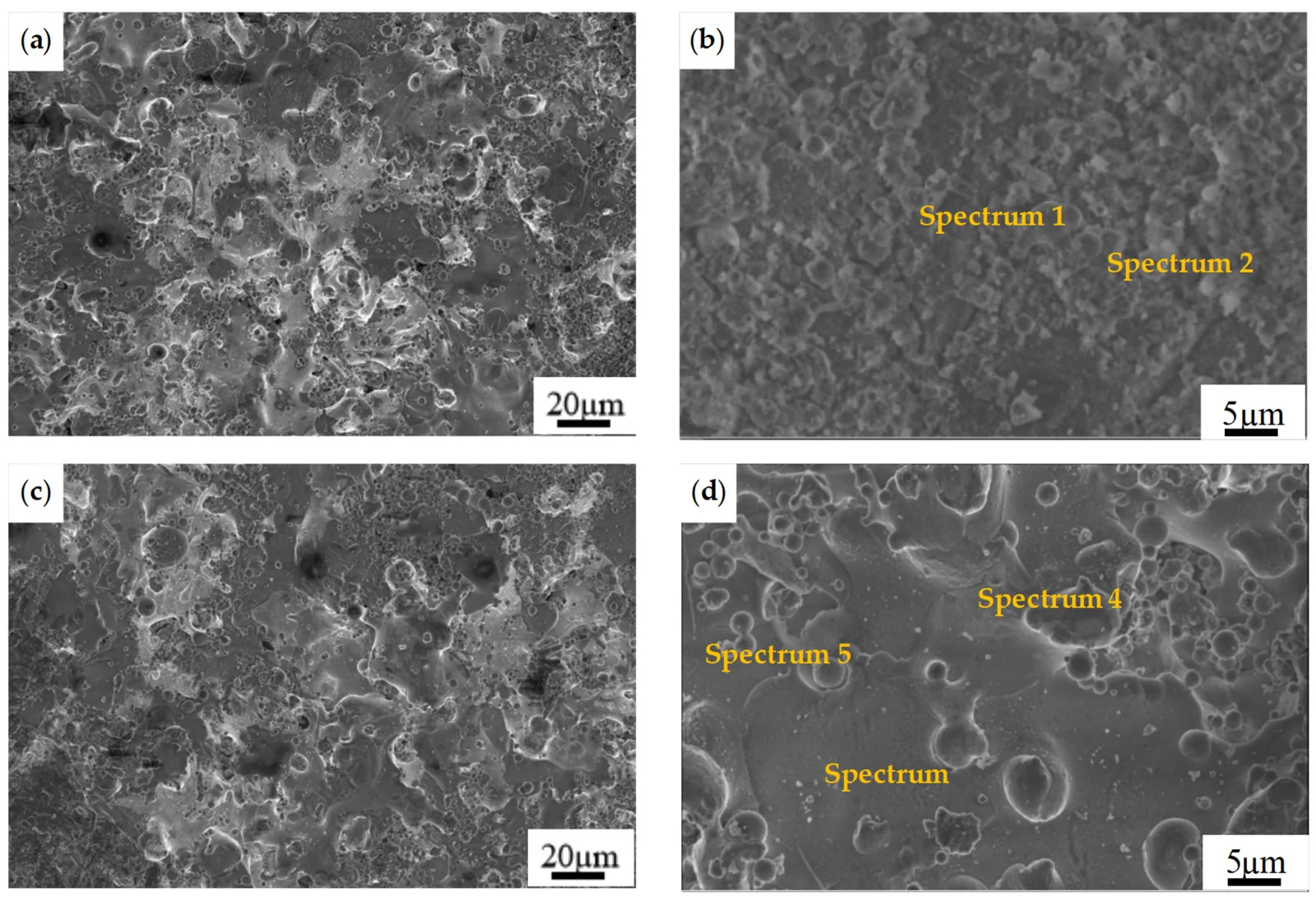

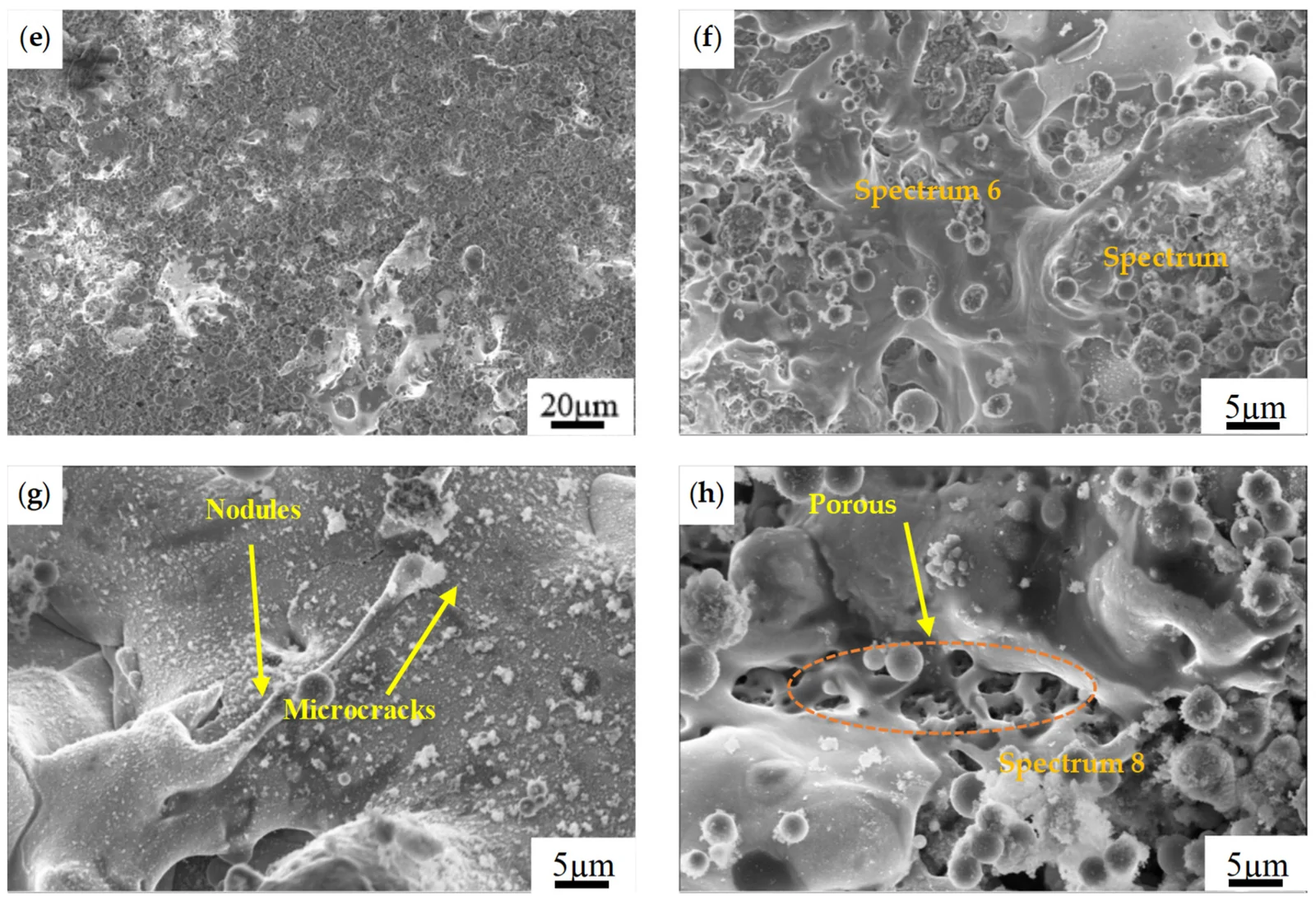
Surface SEM topographies of the composite coatings: (**a**,**b**) H1Z1N; (**c**,**d**) H1Z2N; (**e**,**f**) H1Z3N; (**g**) details of microcracks in H1Z2N; (**h**) network porous structure in H1Z3N.

The cross-sectional micromorphologies of the composite coatings with varying Nb contents deposited on the ZK60 substrate are depicted in Figure 2. All coatings exhibit a dense and continuous structure well-bonded to the magnesium substrate, featuring a characteristic wavy interface without macroscopic delamination or interfacial cracks. Figure 3 illustrates the surface SEM micro-topographies of the H1Z1N, H1Z2N, and H1Z3N coatings at different magnifications. As shown in Figure 3a,b, the H1Z1N coating (10% Nb) displays a well-flattened and stacked splat structure with fluffy, micro-porous features filling the splat boundaries, demonstrating a high degree of particle melting. For the H1Z2N coating (20% Nb), smooth splat stacking is largely preserved, accompanied by minor surface pores and localized microcracks (Figure 3c,d,g). In contrast, increasing the Nb content to 30% (H1Z3N) induces a distinct morphological transition. As observed in Figure 3e,f,h, the fully flattened splat areas decrease, replaced by dispersed, un-melted or partially melted spherical particles and the surface morphology exhibited increased porous characteristics. This porous transition may be related to the combined effects of the high thermal conductivity, high melting point, and particle characteristics of metallic Nb. The increased Nb fraction may promote heat dissipation during spraying, while the relatively high melting point and incomplete melting of Nb particles could reduce splat spreading and contribute to the formation of porous structures.

**Figure 4:**
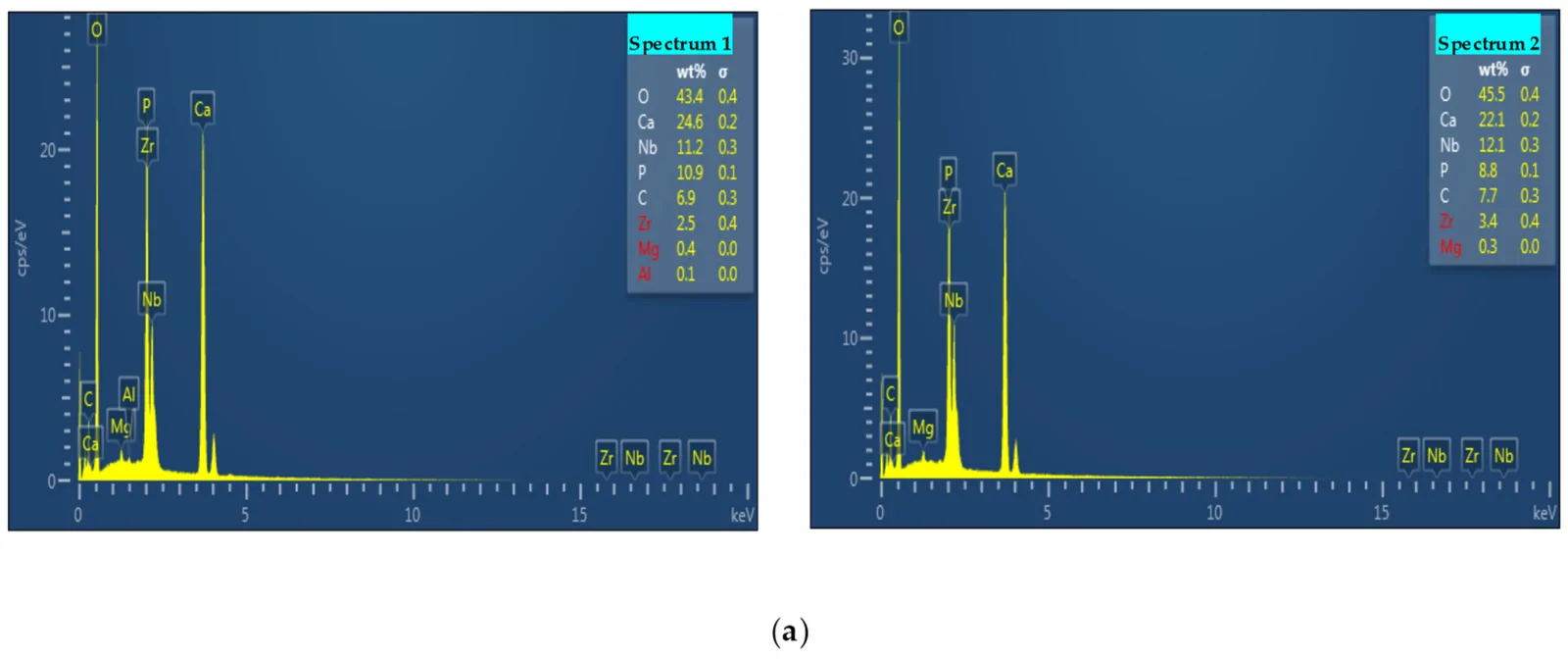

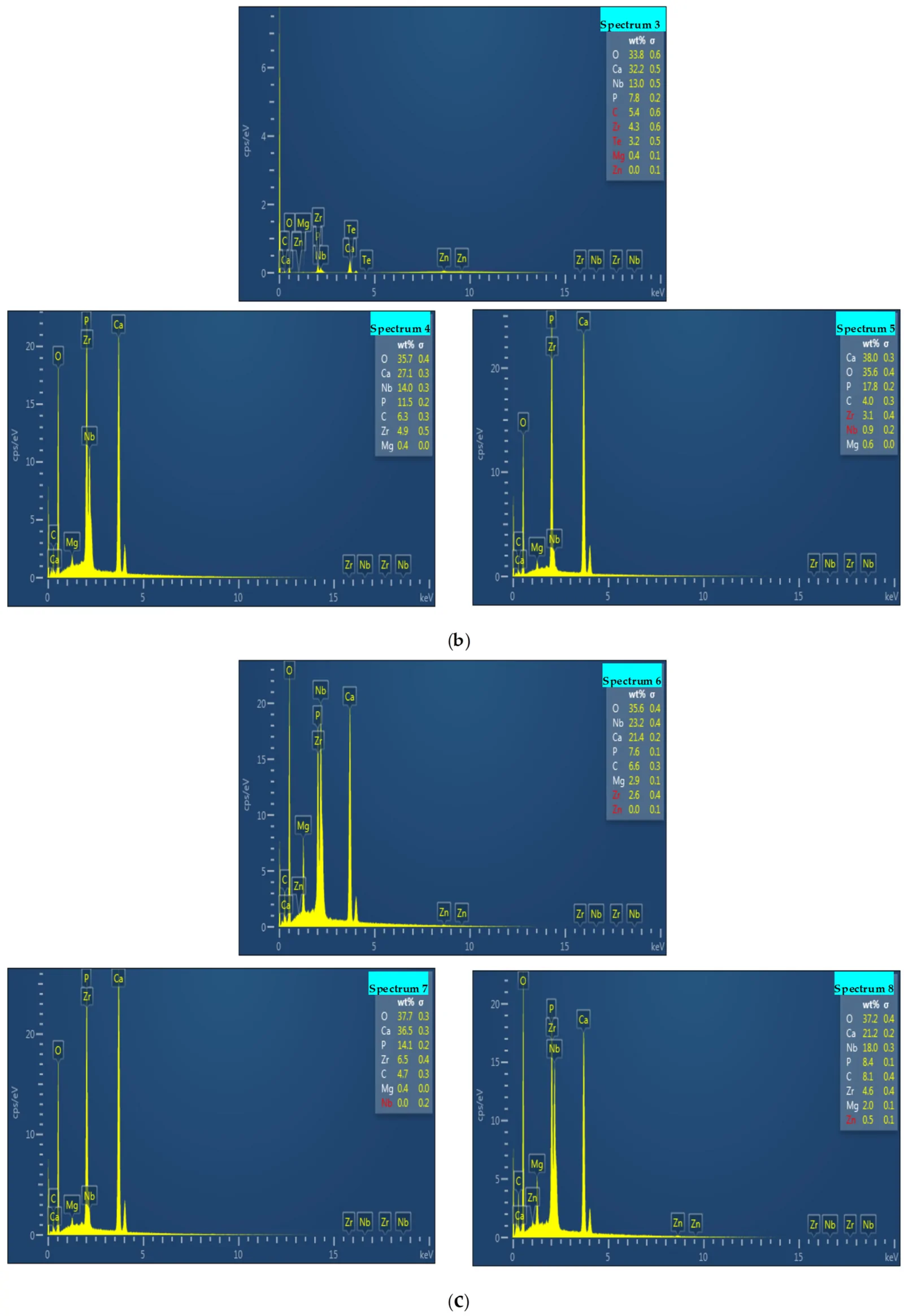
EDS spectra of characteristic surface regions on the composite coatings: (**a**) H1Z1N; (**b**) H1Z2N; (**c**) H1Z3N.

The elemental compositions of characteristic surface regions were further quantified using energy-dispersive X-ray spectroscopy (EDS), as presented in Figure 4. The EDS point analyses detected Ca, P, Zr, and Nb elements in the examined regions, confirming the presence of these elements within the coatings. However, the point measurements cannot fully represent the overall elemental distribution. Notably, localized compositional variations were detected: region 7 in Figure 4c contains Zr but lacks detectable Nb (Ca/P molar ratio ≈ 2.01), whereas region 8 exhibits clear Nb and Zr signals with a Ca/P ratio of 1.95. Overall, increasing the Nb addition to 30% leads to an increase in the overall Ca/P molar ratio of the coating (exceeding that of pure HA). This outcome aligns with the XRD findings, further confirming the accelerated thermal decomposition of HA into calcium-rich phase (TTCP) during high-temperature plasma spraying.

### 3.3. Mechanical and Surface Properties

**Figure 5:**
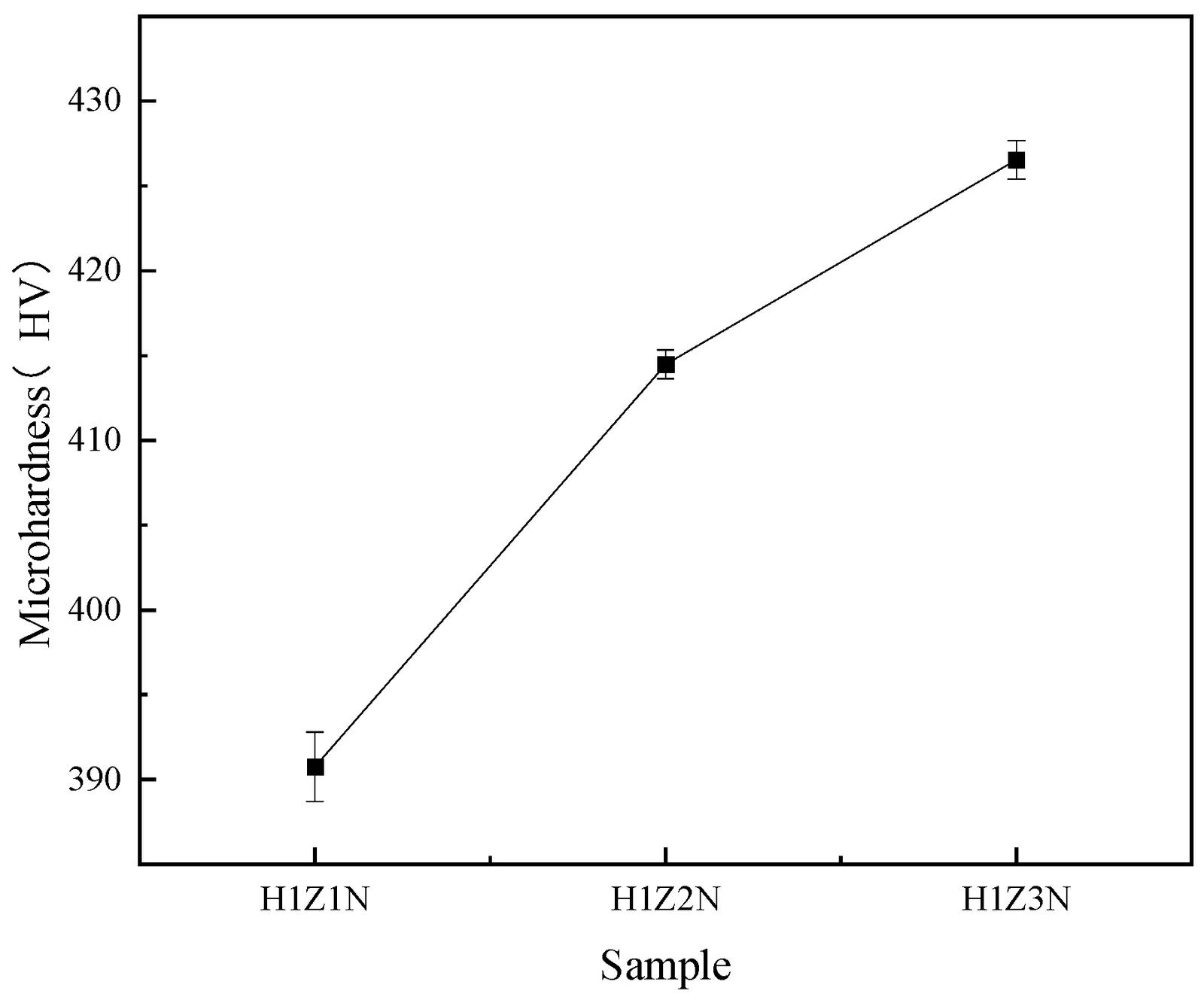
Surface microhardness of composite coatings with different Nb contents. Data are presented as mean ± standard deviation (SD) from three independently prepared specimens.

**Figure 6:**
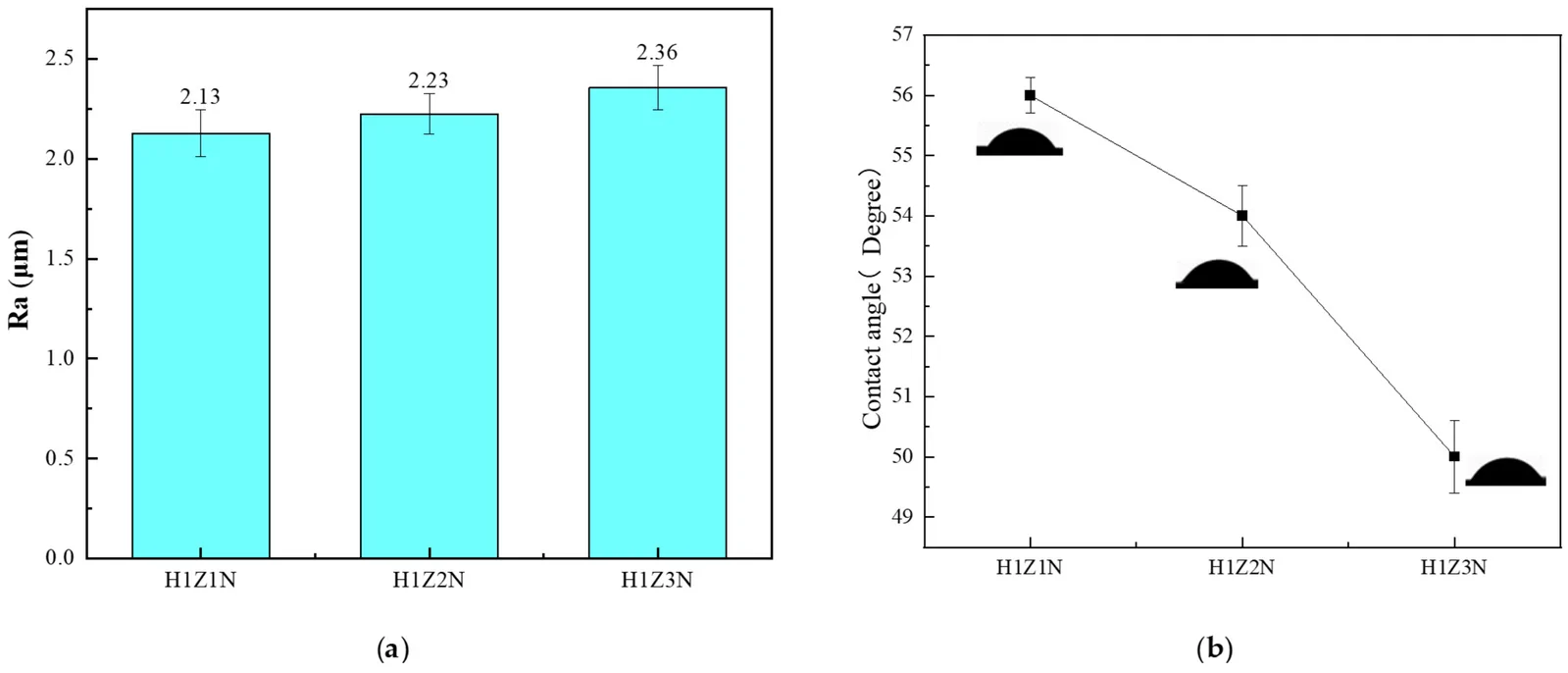
Surface properties of composite coatings with different Nb contents: (**a**) surface roughness; (**b**) water contact angle. Data are presented as mean ± standard deviation (SD) from three independently sprayed specimens.

The microhardness, surface roughness, and wettability of the composite coatings with different Nb contents are presented in Figure 5 and Figure 6. As shown in Figure 5, the surface microhardness of the coatings increases progressively with the addition of metallic Nb. The microhardness values are ~390 HV, ~414 HV, and ~426 HV for H1Z1N, H1Z2N, and H1Z3N coatings, respectively. This increase in microhardness may be associated with the presence of metallic Nb particles and the newly formed Nb_2_O_5_ ceramic phase. However, the growth rate of hardness slows down when the Nb content reaches 30% (H1Z3N only exhibits a 2.9% increase compared to H1Z2N). This plateau occurs because the formation of interconnected porous networks and micro-pores in H1Z3N partially offsets the mechanical reinforcement offered by the added Nb phase. Figure 6 displays the surface roughness and water contact angles of the deposited coatings. As depicted in Figure 6a, the surface roughness increases monotonically with higher Nb content, yielding R_a_ values of 2.128 μm, 2.225 μm, and 2.355 μm for H1Z1N, H1Z2N, and H1Z3N, respectively. Correspondingly, the water contact angle decreases from ~56° (H1Z1N) to ~54° (H1Z2N) and further down to ~50° (H1Z3N), as shown in Figure 6b. The lower contact angle observed for H1Z3N may be associated with its higher surface roughness and porous surface architecture. According to Wenzel’s model, increased surface roughness can enhance the apparent wettability of an intrinsically hydrophilic surface; therefore, these surface features may contribute to the observed decrease in contact angle. According to Wenzel’s model, increased surface roughness amplifies the inherent wettability of a hydrophilic surface, allowing the liquid droplets to spread more effectively into the porous micro-structures. These surface characteristics may also provide favorable conditions for cell attachment; however, their individual contributions were not independently evaluated in the present study.

### 3.4. Corrosion Behavior Analysis

**Figure 7:**
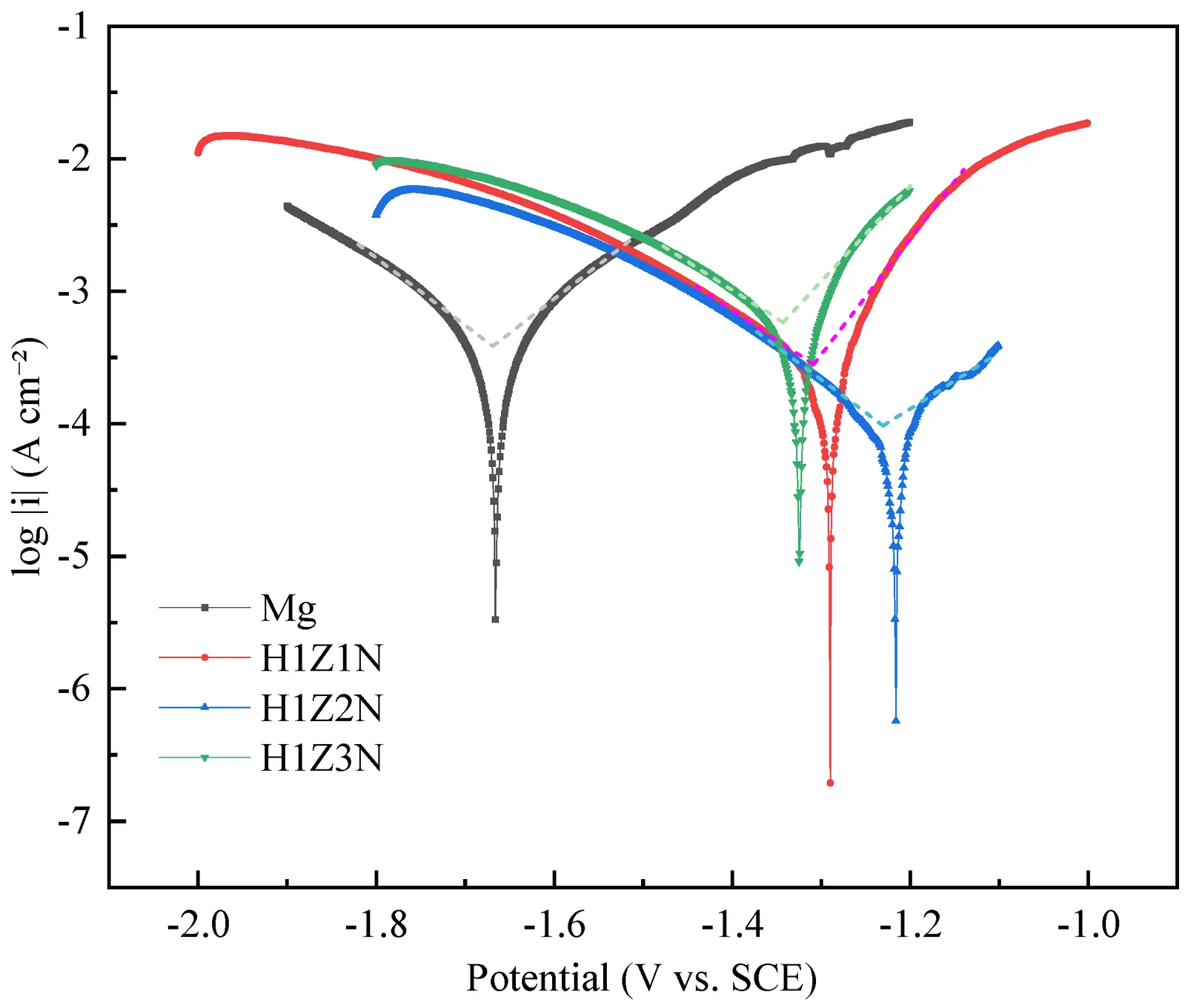
Potentiodynamic polarization curves of the bare ZK60 substrate and composite coatings in SBF solution. Dashed lines indicate the anodic and cathodic linear regression and extrapolation lines.

The electrochemical behavior of the bare ZK60 substrate and composite coatings was evaluated in SBF at room temperature, with polarization curves shown in Figure 7 and parameters listed in Table 2. The electrochemical response varied non-monotonically with increasing Nb content. H1Z2N (20% Nb) exhibited the most positive E_corr_ (−1.230 V) and the lowest I_corr_ (9.688 × 10^−5^ A/cm^2^) among the investigated coatings, suggesting the most favorable electrochemical response under the present testing conditions. H1Z1N exhibited an I_corr_ of 2.798 × 10^−4^ A/cm^2^, which was lower than that of the bare ZK60 substrate (3.850 × 10^−4^ A/cm^2^), whereas H1Z3N showed a higher I_corr_ of 5.833 × 10^−4^ A/cm^2^. The comparatively less favorable electrochemical behavior of H1Z3N may be associated with its interconnected micro-pores and partially melted boundaries, which could facilitate electrolyte access through the coating. In addition, possible galvanic interactions involving Nb-containing regions and electrochemically active phases may also contribute to this behavior, as similar micro-galvanic effects between secondary phases and Mg matrix have been reported in magnesium alloys [43,44]. However, this mechanism requires further confirmation. Since EIS measurements were not performed, the present electrochemical assessment is limited to the polarization-derived E_corr_ and I_corr_ values, and no direct conclusions are drawn regarding coating impedance, barrier properties, or interfacial electrochemical processes.

### 3.5. In Vitro Biocompatibility

**Figure 8:**
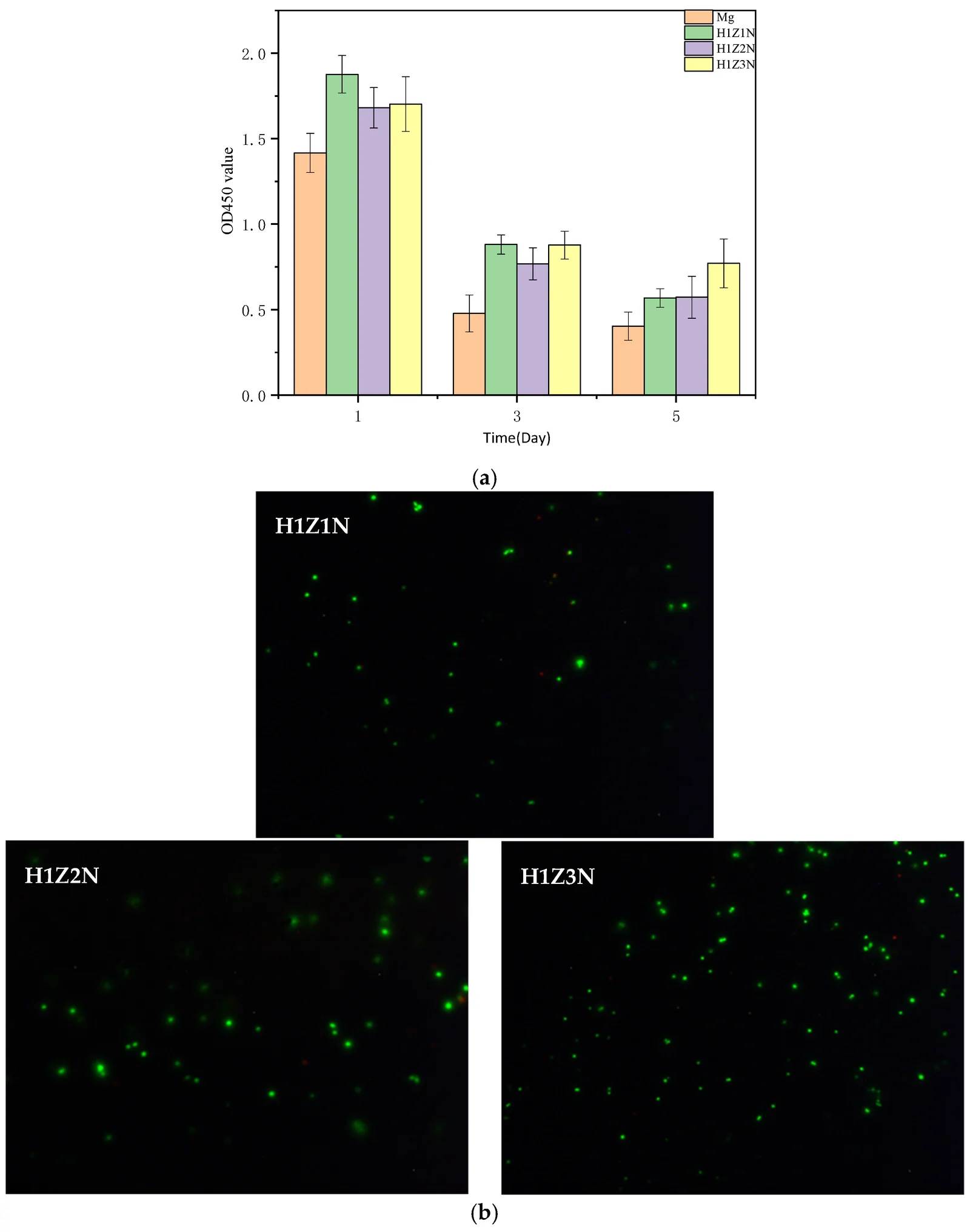
In vitro cytocompatibility evaluation: (**a**) CCK-8 OD450 results after 1, 3, and 5 days of incubation; (**b**) live cell green fluorescence images on H1Z1N, H1Z2N, and H1Z3N coatings on day 5. Data in (**a**) are presented as mean ± standard deviation (SD) from three parallel samples.

The in vitro biocompatibility of the bare ZK60 substrate and composite coatings was evaluated by CCK-8 assay and fluorescence images of attached cells after 1, 3, and 5 days of culture, as shown in Figure 8. As depicted in Figure 8a, the OD450 values of the coated samples were higher than that of the bare Mg substrate across all culture periods. Over time, the OD450 values of all samples gradually decreased, which may be related to Mg degradation and changes in the culture environment. Notably, on day 5, the H1Z3N coating (30% Nb) achieved the highest OD450 value among all group samples. The fluorescence microscopy images in Figure 8b further support this trend, showing more attached green-fluorescent cells with favorable spreading morphology on H1Z3N. The favorable cellular response of H1Z3N may be associated with its higher surface roughness, enhanced hydrophilicity, and interconnected porous surface morphology, which may provide suitable sites for cell attachment. However, these surface characteristics were not independently controlled in the present study, and their individual contributions to the cellular response cannot be distinguished.

## 4. Conclusions

In this study, HA/ZrO_2_/Nb composite coatings with varying Nb contents (10, 20, and 30%) were successfully deposited on ZK60 magnesium alloy substrates via atmospheric plasma spraying. The feedstock phases (HA, ZrO_2_, and Nb) were well preserved after spraying, accompanied by the formation of TTCP and TCP due to the high-temperature decomposition of HA, as well as Nb_2_O_5_ from the in-flight oxidation of Nb. Increasing the Nb content to 30% altered the phase composition and microstructural characteristics of the coating without altering the types of newly generated phases. Microstructurally, a higher Nb content induced a transition from a dense, well-flattened splat structure to an interconnected porous network containing partially melted spherical grains. Concurrently, the surface microhardness increased to ~426 HV (H1Z3N), the surface roughness rose to 2.355 μm, and the water contact angle decreased to ~50°, indicating improved hydrophilicity. Potentiodynamic polarization measurements showed composition-dependent electrochemical behavior among the investigated specimens. H1Z2N (20% Nb) exhibited the most favorable electrochemical response, with E_corr_ = −1.230 V and the lowest I_corr_ of 9.688 × 10^−5^ A/cm^2^. In contrast, H1Z3N (30% Nb) exhibited a higher I_corr_ of 5.833 × 10^−4^ A/cm^2^. Its interconnected porous morphology and possible galvanic interactions may contribute to this behavior. Nevertheless, H1Z3N showed the highest day-5 OD450 value and favorable cell spreading. These results indicate an observed trade-off among the investigated compositions between electrochemical behavior and cellular response.

## Figures and Tables

**Table 1:** Sample designations and weight compositions of the composite powders.

Sample	HA (wt.%)	ZrO_2_ (wt.%)	Nb (wt.%)
H1Z1N	80	10	10
H1Z2N	70	10	20
H1Z3N	60	10	30

**Table 2:** Electrochemical parameters derived from polarization-curve fitting.

Sample	E_corr_ (V vs. SCE)	I_corr_ (A/cm^2^)	β_c_ (mV/dec)	β_a_ (mV/dec)	$R_{c}^{2}$	$R_{a}^{2}$
Mg	−1.669	3.850 × 10^−4^	196.39	193.44	0.99072	0.98961
H1Z1N	−1.309	2.798 × 10^−4^	223.96	114.86	0.99865	0.98989
H1Z2N	−1.230	9.688 × 10^−5^	200.68	237.21	0.99899	0.96352
H1Z3N	−1.343	5.833 × 10^−4^	234.30	138.86	0.99590	0.98894

## Data Availability

The original contributions presented in this study are included in the article. Further inquiries can be directed to the corresponding author.

## References

[1] Gao C., Peng S., Feng P., Shuai C. (2017). Bone biomaterials and interactions with stem cells. Bone Res..

[2] Maksimkin A.V., Senatov F.S., Anisimova N.Y., Kiselevskiy M.V., Zalepugin A.D. (2017). Multilayer porous UHMWPE scaffolds for bone defects replacement. Mater. Sci. Eng. C.

[3] Nuss K.M., von Rechenberg B. (2008). Biocompatibility issues with modern implants in bone-a review for clinical orthopedics. Open Orthop. J..

[4] Liu C., Ren Z., Xu Y., Pang S., Zhao X., Zhao Y. (2018). Biodegradable Magnesium Alloys Developed as Bone Repair Materials: A Review. Scanning.

[5] Matsuno H., Yokoyama A., Watari F., Uo M., Kawasaki T. (2001). Biocompatibility and osteogenesis of refractory metal implants, titanium, hafnium, niobium, tantalum and rhenium. Biomaterials.

[6] Zheng Y.F., Gu X.N., Witte F. (2014). Biodegradable metals. Mater. Sci. Eng. R Rep..

[7] Kamrani S., Fleck C. (2019). Biodegradable magnesium alloys as temporary orthopaedic implants: A review. Biometals.

[8] Hornberger H., Virtanen S., Boccaccini A.R. (2012). Biomedical coatings on magnesium alloys—A review. Acta Biomater..

[9] Witte F. (2010). The history of biodegradable magnesium implants: A review. Acta Biomater..

[10] Thakur B., Barve S., Pesode P. (2022). Magnesium-Based Nanocomposites for Biomedical Applications. Advanced Materials for Biomechanical Applications.

[11] Yang H., Xia K., Wang T., Niu J., Song Y., Xiong Z., Zheng Y. (2016). Growth, in vitro biodegradation and cytocompatibility properties of nano-hydroxyapatite coatings on biodegradable magnesium alloys. J. Alloys Compd..

[12] Nassif N., Ghayad I. (2013). Corrosion Protection and Surface Treatment of Magnesium Alloys Used for Orthopedic Applications. Adv. Mater. Sci. Eng..

[13] Chen K., Dai J., Zhang X. (2015). Improvement of corrosion resistance of magnesium alloys for biomedical applications. Corros. Rev..

[14] Dunne C.F., Levy G.K., Hakimi O., Warnke P.H., Morrell A.P., Labeed O.S. (2016). Corrosion behaviour of biodegradable magnesium alloys with hydroxyapatite coatings. Surf. Coat. Technol..

[15] Yang J., Cui F.Z., Lee I.S. (2011). Surface Modifications of Magnesium Alloys for Biomedical Applications. Ann. Biomed. Eng..

[16] Chen J., Yang Y., Etim I.P., Huang C., Han J. (2021). Recent Advances on Development of Hydroxyapatite Coating on Biodegradable Magnesium Alloys: A Review. Materials.

[17] Chen H., Wang Y., He L., Zhang X., Mei Y., Wu T., Wang J., Zheng Y., Tang H. (2025). Surface Engineering of Biodegradable Magnesium Alloys as Orthopedic Implant Materials: Recent Developments and Future Prospects. Coatings.

[18] Kumar G.S., Lalithambigai K., Van Minh N., Shkir M., Sayed M.A. (2025). Hydroxyapatite coatings for biodegradable magnesium alloys: Recent advances, strategies, challenges, and future prospects. J. Magnes. Alloys.

[19] Nabiyouni M., Bruckner T., Zhou H., Gbureck U., Bhaduri S.B. (2018). Magnesium-based bioceramics in orthopedic applications. Acta Biomater..

[20] Mahapatro A., Arshanapalli S.A. (2017). Bioceramic Coatings on Magnesium Alloys. J. Bio-Tribo-Corros..

[21] Cao H., Liu X. (2013). Plasma-Sprayed Ceramic Coatings for Osseointegration. Int. J. Appl. Ceram. Technol..

[22] Sun L., Berndt C.C., Grey C.P. (2003). Phase, structural and microstructural investigations of plasma sprayed hydroxyapatite coatings. Mater. Sci. Eng. A.

[23] Sun L., Berndt C.C., Gross K.A., Kucuk A. (2001). Material fundamentals and clinical performance of plasma-sprayed hydroxyapatite coatings: A review. J. Biomed. Mater. Res..

[24] Tancred D., McCormack B.A.O., Carr A.J. (1998). A quantitative study of the sintering and mechanical properties of hydroxyapatite/phosphate glass composites. Biomaterials.

[25] Zhang Q., Chen J., Feng J., Cao Y., Deng C., Zhang X. (2003). Dissolution and mineralization behaviors of HA coatings. Biomaterials.

[26] Liu X.M., He D.Y., Zhou Z., Guo H., Wang X.Y. (2018). Atmospheric Plasma-Sprayed Hydroxyapatite Coatings with (002) Texture. J. Therm. Spray Technol..

[27] Kumar P., Kudva S.A., Anilkumar T., Ramesh S., Ramesh M.R., Prabhu A., Anne G. (2025). Synthesis and characteristics of Fe/Ni/Cr oxide nanoparticles/PLA hybrid composite coatings on Mg-Zn-Ca alloy. J. Mater. Res. Technol..

[28] Feng L., Wu Z., Huang Y., Shen L., Qiao B., Wu J., Wen N., Hu J., Deng B. (2026). Degradation behavior, osteogenesis, and antimicrobial properties of Ga-coated ZK60 Mg alloys for medical implants. J. Magnes. Alloys.

[29] Yugeswaran S., Yoganand C.P., Kobayashi A., Paraskevopoulos K.M., Subramanian B. (2012). Mechanical properties, electrochemical corrosion and in-vitro bioactivity of yttria stabilized zirconia reinforced hydroxyapatite coatings prepared by gas tunnel type plasma spraying. J. Mech. Behav. Biomed. Mater..

[30] Fu L., Khor K.A., Lim J.P. (2001). Processing, microstructure and mechanical properties of yttria stabilized zirconia reinforced hydroxyapatite coatings. Mater. Sci. Eng. A.

[31] Patil N.A., Kandasubramanian B. (2020). Biological and mechanical enhancement of zirconium dioxide for medical applications. Ceram. Int..

[32] Silva V.V., Domingues R.Z., Lameiras F.S. (2001). Microstructural and mechanical study of zirconia-hydroxyapatite (ZH) composite ceramics for biomedical applications. Compos. Sci. Technol..

[33] O’Brien B., Stinson J., Carroll W. (2008). Development of a new niobium-based alloy for vascular stent applications. J. Mech. Behav. Biomed. Mater..

[34] Wei P., Fang J., Fang L., Wang K., Lu X., Ren F. (2019). Novel niobium and silver toughened hydroxyapatite nanocomposites with enhanced mechanical and biological properties for load-bearing bone implants. Appl. Mater. Today.

[35] Verma C., Lee S.-Y., Gautam J., Dubey S., Singh P., Rhee K.Y., Ebenso E.E., Alfantazi A., Park S.-J. (2025). Hydroxyapatite-based coatings for corrosion resistance and self-healing in biomedical and industrial applications. Acta Biomater..

[36] Fathi M.H., Salehi M., Mortazavi V., Mousavi S.B., Parsapour A. (2006). Novel hydroxyapatite/niobium surface coating for endodontic dental implant. Surf. Eng..

[37] Nagarajan S., Raman V., Rajendran N. (2010). Synthesis and electrochemical characterization of porous niobium oxide coated 316L SS for orthopedic applications. Mater. Chem. Phys..

[38] Karlinsey R.L., Hara A.T., Yi K., Mackey A.C. (2006). Bioactivity of novel self-assembled crystalline Nb_2_O_5_ microstructures in simulated and human salivas. Biomed. Mater..

[39] Wan X., Fang S., Xu S., Yu L., Zhou J., Qian S., Huang F., Ma C. (2024). Enhanced Anti-Corrosion and Biological Performance of Plasma-Sprayed Nb/ZrO_2_/HA Coatings on ZK60 Mg Alloy. Coatings.

[40] Singh B., Singh G., Sidhu B.S., Bhatia N. (2019). In-vitro assessment of HA-Nb coating on Mg alloy ZK60 for biomedical applications. Mater. Chem. Phys..

[41] Sun S., Li J., Gu H., Xu G., Ge R. (2026). Study on the Microstructure and Properties of Nb/ZrO_2_/HA Composite Coatings by Plasma Spraying Process Parameters. Coatings.

[42] (2019). Biological Evaluation of Medical Devices—Part 15: Identification and Quantification of Degradation Products from Metals and Alloys.

[43] Xu L., Liu X., Sun K., Fu R., Wang G. (2022). Corrosion Behavior in Magnesium-Based Alloys for Biomedical Applications. Materials.

[44] Song G., Atrens A. (2003). Understanding magnesium corrosion—A framework for improved alloy performance. Adv. Eng. Mater..

